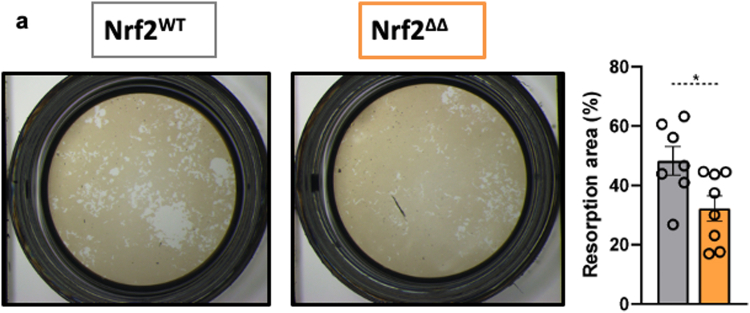# Nrf2 dynamically regulates RANKL-induced osteoclastogenesis and cathepsin K function

**DOI:** 10.1016/j.jbc.2026.113263

**Published:** 2026-06-19

**Authors:** Cesar A. Speck-Hernandez, Taíssa C. de Souza Furtado, Laisa Y. de Souza, Keteryne R. da Silva, Gabriel V. Lucena Silva, Luis Gonzalez Osuna, Gabriel A. Publio, Ayda H. Schneider, Paulo H. Melo, Gabriella Scalice-Chiari, Jose Carlos Alves-Filho, Fernando de Queiroz Cunha, Thiago Mattar Cunha, Sandra Yasuyo Fukada

**Affiliations:** 1 Center for Research in Inflammatory Diseases, Ribeirao Preto Medical School, University of Sao Paulo, Ribeirao Preto, Brazil; 2 Department of Pharmacology, Ribeirao Preto Medical School, University of Sao Paulo, Ribeirao Preto, Brazil; 3 Department of Biomolecular Sciences, Laboratory of Bone Biology, School of Pharmaceutical Sciences, Ribeirão Preto, University of São Paulo, Ribeirão Preto, Brazil

**Keywords:** osteoclasts, Nrf2, ROS, mitochondria, lipid peroxidation, oxidative stress

## Abstract

Osteoclasts mediate bone resorption primarily through the protease Cathepsin K. RANKL, the master cytokine driving osteoclastogenesis, elevates reactive oxygen species (ROS) levels that promote osteoclast differentiation; however, excessive ROS can lead to oxidative stress and cellular damage. To counteract the detrimental effects of ROS, osteoclasts activate antioxidant defense mechanisms, including the NRF2 pathway. Here, we identify that antioxidant responses are dynamically regulated during osteoclastogenesis and osteoclast activation. Through a combined bioinformatic and genetic approach using engineered mouse models, we demonstrate a dual role of RANKL in regulating antioxidant responses in osteoclasts: while it suppresses glutathione-mediated antioxidant defenses, RANKL activates Nrf2-dependent mechanisms during osteoclast differentiation. Genetic deletion of *Nrf2* (*Nfe2l2*) *in vitro* enhances osteoclast formation, whereas impairs osteoclast resorptive function, reducing cathepsin K activity. Nrf2-deficient osteoclasts exhibit increased lipid peroxidation, mitochondrial dysfunction, and lysosomal instability without alterations in cell viability. Together, these findings identify NRF2 as a critical regulator of osteoclast function, essential for maintaining redox balance and lysosomal integrity during bone resorption. This study reveals an intricate interplay between RANKL-induced oxidative signaling and antioxidant regulation, highlighting NRF2 as a critical determinant of osteoclast-mediated bone resorption.

Osteoclasts are multinucleated cells derived from the monocyte-macrophage lineage, responsible for bone resorption, actively degrading the bone matrix. Their differentiation is regulated by macrophage colony-stimulating factor (M-CSF) and receptor activator of nuclear factor-κB ligand (RANKL) (1). Osteoclast activity is crucial for healthy skeletal maintenance, primarily by removing damaged bone during skeletal remodeling. Cathepsin K is the main protease released by osteoclasts to degrade type I collagen (2). The genetic deletion or functional alteration of Cathepsin K leads to impaired osteoclastic bone resorption, resulting in osteopetrosis, an abnormal bone mineralization condition (3). In osteoporosis, osteoclasts exhibit an increased bone degradation activity, causing bone fragility (4).

The osteoclast differentiation is driven by RANK-RANKL signaling (5), which activate downstream proteins such as NF-kB, p38-MAPK, and JNK, leading to the expression of key transcription factors including AP-1, c-Jun, c-Fos, and NFATc1 (6). The activation of these pathways is accompanied by a significant increase in ROS sub-products, which act as a crucial molecular signal during differentiation (7). Sustained or excessive ROS levels can lead to oxidative stress, damaging lipids, proteins, organelles, and DNA (8). To counteract this, osteoclasts must have an efficient antioxidant defense program to mitigate the detrimental effects of excessive ROS production. The transcription factor BFR2 plays a crucial role in this process by inducing the expression of several proteins associated with antioxidant responses (9). Studies have explored the impact of NRF2 pathway on osteoclast differentiation and resorptive functions (10). Although previous studies have shown that the deletion of *Nrf2* enhances osteoclast differentiation through elevated ROS levels (11), the functional consequences of *Nrf2* loss on mature osteoclast activity remain poorly understood.

In this study, we demonstrate that RANKL-induced osteoclastogenesis is dynamically regulated by NRF2 in a stage-dependent manner. Through transcriptomic profiling and conditional knockout models, we show that *Nrf2* deficiency enhances osteoclast formation but paradoxically impairs bone resorptive function, as evidenced by reduced cathepsin K expression and resorptive activity. Furthermore, we provide evidence that *Nrf2* deficiency leads to oxidative damage, including lipid peroxidation, mitochondrial dysfunction, and lysosomal instability, ultimately compromising osteoclast function. Together, these findings identify *Nrf2* as a temporal and functional regulator of osteoclast biology that uncouples differentiation from resorptive capacity by maintaining redox and organelle integrity.

## Results

### RANKL transiently regulates oxidative stress during early osteoclast differentiation

**Figure 1:**
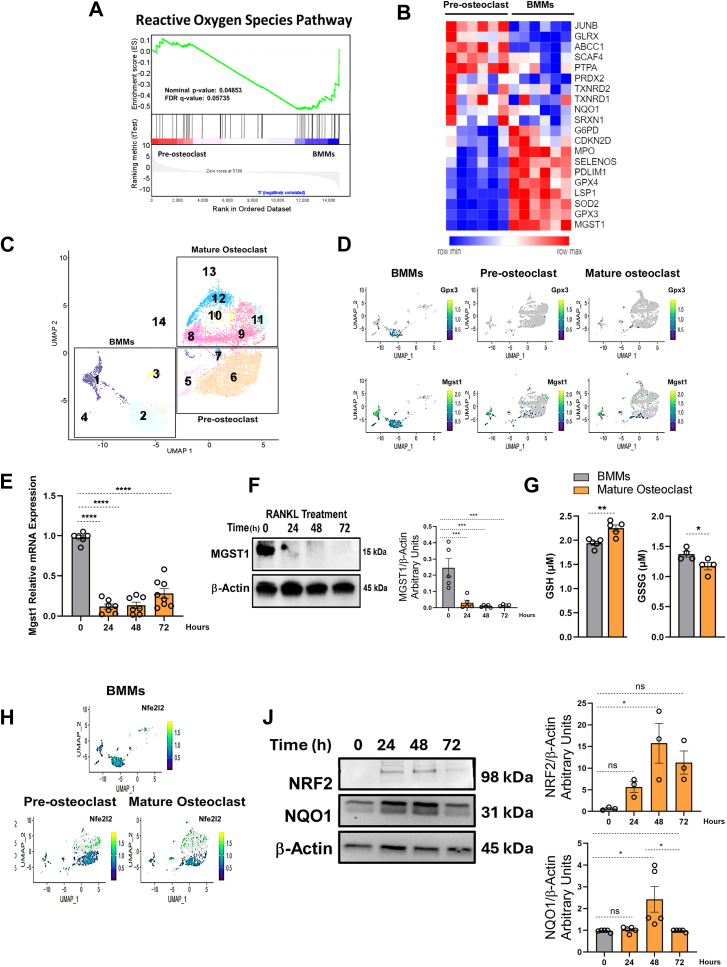
**RANKL treatment reduces glutathione-mediated antioxidant responses while simultaneously activating Nrf2.***A*, gene set enrichment analysis (GSEA) of the Reactive Oxygen Species (ROS) pathway comparing pre-osteoclasts with untreated bone marrow macrophages (BMMs). *B*, heatmap of genes associated with the ROS pathway comparing pre-osteoclasts (24 h) with untreated BMMs (0 h). *C*, uniform Manifold Approximation and Projection (UMAP) visualization of 14 clusters from the osteoclast culture system, categorized as BMMs, pre-osteoclasts, and mature osteoclasts. *D*, UMAP visualization of *Gpx3* and *Mgst1* gene expressions in BMMs, pre-osteoclasts, and mature osteoclasts. *E*, relative expression of *Mgst1* during osteoclast differentiation. The graph summarizes two independent experiments (n = 8). Data are presented as mean ± SEM. One-way ANOVA was used to calculate *p* values. ∗∗∗∗*p* < 0.0001. *F*, Western blot of MGST1 and β-actin expression levels in BMM treated with RANKL at different time points (0, 24, 48, and 72 h). Bar graphs summarize three independent experiments. Data are presented as mean ± SEM. One-way ANOVA was used to calculate *p* values. ∗∗∗*p* < 0.001. *G*, fluorometric quantification of reduced glutathione (GSH) and oxidized glutathione (GSSG) in untreated BMMs (0h) and mature osteoclasts (72 h). The graph summarizes two independent experiments (n = 4–5). Data are presented as mean ± SEM. An unpaired two-tailed Student’s *t* test was used to calculate *p* values. ∗*p* < 0.05, ∗∗*p* < 0.01. *H*, UMAP visualization of *Nfe2l2* gene expression during in BMMs, pre-osteoclasts, and mature osteoclasts. *I*, Western blot comparing NRF2 (n = 3), NQ01 (n = 5), and β-actin expression levels in cells treated with RANKL over different time points (0, 24, 48, and 72 h). The blot is representative of 3 independent experiments. Bar graphs summarize 3 independent experiments. Data are presented as mean ± SEM. One-way ANOVA was used to calculate *p* values. ∗*p* < 0.05; ns, not significant.

**Figure S1:**
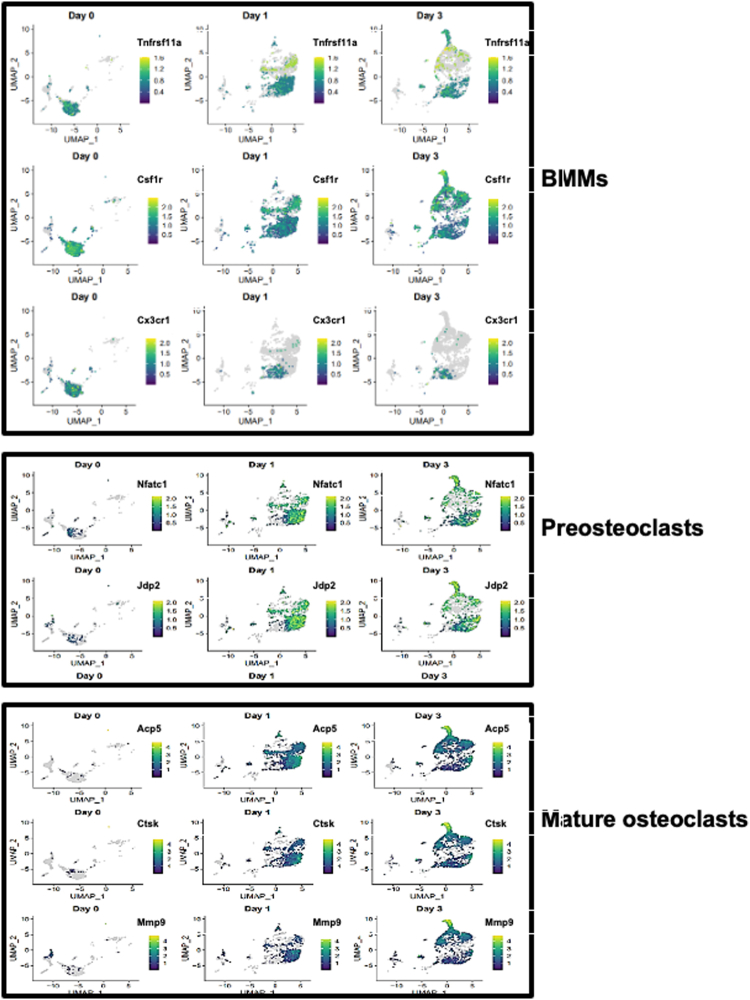


Activation of the RANK/RANKL pathway rapidly induces ROS production during osteoclast differentiation (12), leading us to hypothesize that RANKL promotes osteoclastogenesis, at least in part, by suppressing cellular antioxidant defenses. To test this, we reanalyzed a public bulk RNA-seq dataset (GSE176265) profiling bone marrow macrophages (BMMs) and early RANKL-stimulated osteoclast precursors (13). The comparison of BMMs with pre-osteoclasts (BMMs treated with RANKL for 24 h) revealed a significant downregulation of the “reactive oxygen species (ROS) pathway” gene set in pre-osteoclasts (*p* = 0.04, FDR = 0.05) (Fig. 1*A*). Analysis of individual genes of this pathway showed that several genes associated with antioxidant responses, particularly those related to glutathione metabolism, such as *Gpx3* (glutathione peroxidase 3), *Gpx4* (glutathione peroxidase 4), and *Mgst1* (microsomal glutathione S-transferase 1) were significantly downregulated in pre-osteoclasts (Fig. 1*B*). We further evaluated a public database GSE147174 containing single-cell RNA-seq data (14) that combined transcriptomic analyses comparing bone marrow macrophages (BMMs), pre-osteoclast (day 1 of RANKL) and mature osteoclast (day 3 of RANKL). We identified 14 clusters (Fig. 1*C*) and grouped them based on specific osteoclast markers. Clusters 1, 2, 3 and 4 were identified as BMMs, clusters 5, 6, and 7 as preosteoclasts, and clusters 9 to 13 as mature osteoclasts (Fig. S1, *A*–*C*). The expression of *Gpx3 and Mgst1* declined in pre-osteoclasts and mature osteoclasts compared to BMMs (Fig. 1*D*).

We then validated the RNA-seq findings by quantifying *Mgst1* expression using quantitative real-time PCR and Western blot. Our data confirms that RANKL markedly reduced *Mgst1* mRNA and MGST1 protein levels during osteoclast differentiation (Fig 1, *E* and *F*). Because MGST1 is a key regulator of glutathione metabolism, we next evaluated the cellular redox status by measuring the intracellular GSH and oxidized GSH (GSSG) levels in BMMs and mature osteoclasts. Mature osteoclasts exhibited increased GSH and reduced levels of GSSG (Fig. 1*G*), indicating a less oxidative intracellular environment. The shift toward a more reduced intracellular environment is consistent with the establishment of a stronger antioxidant response in mature osteoclasts, despite the downregulation of several antioxidant related genes at the preosteoclast stage.

In contrast, transcriptomic analyses revealed that *Nfe2l2* (Nrf2) expression is upregulated during the early stage of RANKL-induced pre-osteoclasts differentiation and slightly declined in mature osteoclasts (Fig. 1*H*). Consistent with these findings, NRF2 protein levels transiently increased at 24 and 48 h after RANKL stimulation, followed by a marked reduction at 72 h, corresponding to mature osteoclasts. In parallel, the expression of NQ01, a canonical NRF2 downstream target gene, closely mirrored the temporal pattern of NRF2, showing increased levels at 24 and 48 h and a reduction at 72 h (Fig. 1*I*). These findings suggest that NRF2 plays a stage-specific role in osteoclastogenesis, providing transient oxidative protection during early osteoclast differentiation, while in fully mature osteoclasts, it appears to be less critical for maintaining the redox balance.

### Nrf2 deficiency enhances osteoclast differentiation but impairs cathepsin K expression and bone resorption

**Figure 2:**
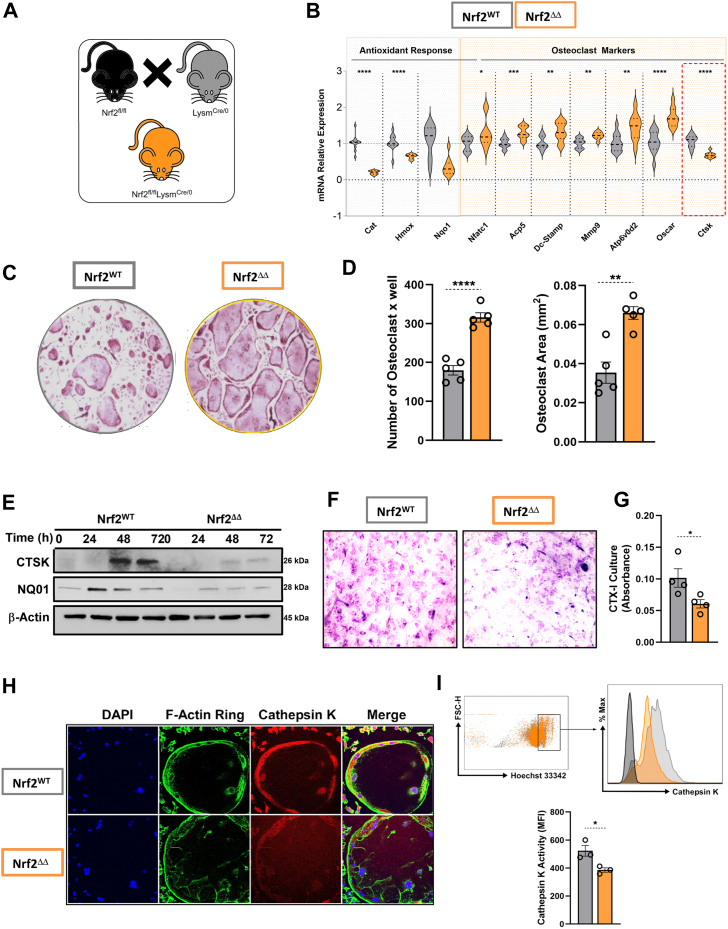
**Deleting Nrf2 in pre-osteoclasts impairs Cathepsin K production and bone matrix degradation.***A*, breeding strategy in which Nrf2^fl/fl^ animals were crossed with LysM^Cre^ animals to generate Nrf2^fl/fl^ LysM^Cre^.unknown. *B*, relative mRNA expression of antioxidant genes (*left, gray*) and mature osteoclast differentiation markers (*right, orange*) after 3 days of culture (72 h of treatment with M-CSF, 50 ng/ml, and RANKL, 10 ng/ml). Violin plots represent two independent experiments (n = 6). Data are presented as mean ± SEM. Unpaired two-tailed Student’s *t* test was used to calculate *p* values. ∗*p* < 0.05, ∗∗*p* < 0.01, ∗∗∗*p* < 0.001, ∗∗∗∗*p* < 0.0001. *C*, TRAP staining of mature osteoclasts from Nrf2^wt^ (*gray*) and Nrf2^ΔΔ^ (*orange*) animals. *D*, quantification of osteoclast number and area per well. Bar graphs summarize two independent experiments (n = 5). Data are presented as mean ± SEM. Unpaired two-tailed Student’s *t* test was used to calculate *p* values. ∗∗*p* < 0.01, ∗∗∗∗*p* < 0.0001. *E*, Western blot comparing CTSK (n = 3) and NQ01 (n = 4) protein levels during osteoclast differentiation in Nrf2^wt^ (*gray*) and Nrf2^ΔΔ^ (*orange*) animals. β-actin was used as a loading control. Blots are representative of three independent experiments. *F*, toluidine blue staining of bovine cortical bone discs showing degradation by mature osteoclasts from Nrf2^wt^ (*gray*) and Nrf2^ΔΔ^ (*orange*) animals. Images are representative of two independent experiments. *G*, ELISA quantification of CTX-I in the culture supernatant of osteoclasts cultured on bovine cortical bone discs. The bar graph summarizes two independent experiments (n = 4). Data are presented as mean ± SEM. Unpaired two-tailed Student’s *t* test was used to calculate *p* values. ∗*p* < 0.05. *H*, confocal microscopy (60 × ) of osteoclasts from Nrf2^wt^ (*gray*) and Nrf2^ΔΔ^ (*orange*) animals treated with the Magic *Red* Cathepsin K probe and phalloidin-A488. Images are representative of two independent experiments. *I*, flow cytometry of mature osteoclasts labeled with Magic Red Cathepsin K and Hoechst 33,342. The bar graph summarizes two independent experiments (n = 3). Data are presented as mean ± SEM. Unpaired two-tailed Student’s *t* test was used to calculate *p* values. ∗*p* < 0.05.

To investigate the functional role of Nrf2 on osteoclast differentiation, we employed BMMs from Nrf2^fl/fl^ Lysm^Cre/0^ mice (Nrf2^ΔΔ^) (Fig. 2*A*) and Lysm^Cre/0^ mice (Nrf2^WT^) to delete Nrf2 in pre-osteoclasts. Osteoclasts lacking Nrf2 exhibited reduced expression of key antioxidant enzymes, including *Cat, Hmox1*, and *Nqo1* (Fig. 2*B*), suggesting that these genes are downstream targets of NRF2 and part of the NRF2-dependent antioxidant response. Simultaneously, we observed an upregulation of several osteoclast marker genes (*Nfatc1*, *Acp5*, *Dcstamp, Mmp9*, *Atp6v0d2*, and *Oscar*) (Fig. 2*B*) in Nrf2-deficient group compared to control, which was synchronized with enhanced osteoclast differentiation, characterized by increased size and multinucleation (Fig. 2, *C* and *D*). Intriguingly, Nrf2-deficient osteoclasts displayed significantly reduced expression of Ctsk both mRNA (Fig. 2*B*) and protein levels (Fig. 2*E*) when compared to control, which was associated with the reduced level of NQ01 protein in Nrf2^ΔΔ^ cells (Fig. 2*E*). Our data shows that the deficiency of Nrf2 also impaired the ability of osteoclasts to degrade mineral compounds in a hydroxyapatite-coated plate (Fig. S2*A*). Moreover, bone resorption assay demonstrated that Nrf2^ΔΔ^ osteoclasts exhibit a reduced bone degradation area in bone slice and reduced level of CTX-1 compared with control (Fig. 2, *F* and *G*), which was associated with a significant reduction in cathepsin K activity in Nrf2-deficient osteoclasts (Fig. 2, *H* and *I*). Together, these results demonstrate that although the loss of Nrf2 in pre-osteoclasts improves osteoclastogenesis, it impairs the assembly of functional resorptive machinery, particularly the production of CTSK by mature osteoclasts.

### The deletion of Nrf2 in preosteoclasts increases ROS production and disrupts mitochondrial homeostasis

**Figure 3:**
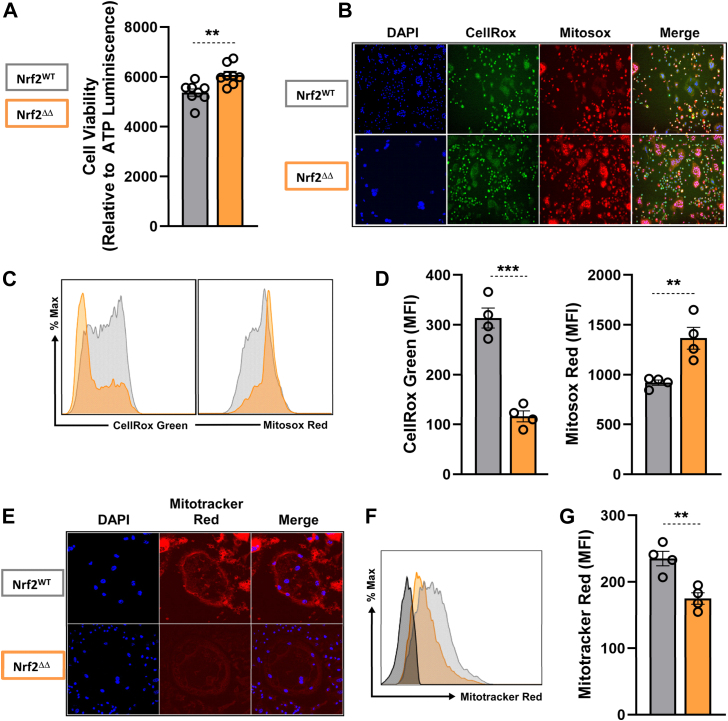
**Nrf2-deficient osteoclasts lose mitochondrial stability and experience severe oxidative stress.***A*, quantification of cell viability using an ATP-luminescence assay in mature osteoclasts from Nrf2^wt^ (*gray*) and Nrf2^ΔΔ^ (*orange*) animals. Bar graph summarizes two independent experiments (n = 8). Data are presented as mean ± SEM. Unpaired two-tailed Student’s *t* test was used to calculate *p* values. ∗∗*p* < 0.01. *B*, high-content microscopy analysis of mature osteoclasts Nrf2^wt^ (*gray*) and Nrf2^ΔΔ^ (*orange*) animals labeled with CellROX and MitoSOX Red probes. Images are representative of two independent experiments. *C*, flow-cytometric analysis of mature osteoclasts from Nrf2^wt^ (*gray*) and Nrf2^ΔΔ^ (*orange*) animals quantifying CellROX and MitoSOX Red fluorescence. Histograms represent two independent experiments. *D*, median fluorescence intensity (MFI) of MitoSOX Red and CellROX signals in mature osteoclasts from Nrf2^wt^ (*gray*) and Nrf2^ΔΔ^ (*orange*) animals. Bar graph summarizes two independent experiments (n = 4). Data are presented as mean ± SEM. Unpaired two-tailed Student’s *t* test was used to calculate *p* values. ∗∗*p* < 0.01, ∗∗∗*p* < 0.001. *E*, confocal microscopy (60 × ) of mature osteoclasts labeled with MitoTracker Red from Nrf2^wt^ (*gray*) and Nrf2^ΔΔ^ (*orange*) animals. Images are representative of two independent experiments. *F*, flow-cytometric analysis of mitochondrial density and membrane potential using MitoTracker Red in osteoclasts from Nrf2^wt^ (*gray*) and Nrf2^ΔΔ^ (*orange*) animals. Plots are representative of two independent experiments. *G*, median fluorescence intensity (MFI) of MitoTracker Red in mature osteoclasts from Nrf2^wt^ (*gray*) and Nrf2^ΔΔ^ (*orange*) animals. Bar graph summarizes two independent experiments (n = 4). Data are presented as mean ± SEM. Unpaired two-tailed Student’s *t* test was used to calculate *p* values. ∗∗*p* < 0.01.

We next investigated the effect of Nrf2 deletion on the oxidative and metabolic profile of osteoclasts. Using an ATP-fluorescent assay, we found that Nrf2^ΔΔ^ osteoclasts exhibit higher metabolic activity (ATP production) compared to Nrf2^WT^ osteoclasts (Fig. 3*A*). High-content imaging and flow cytometry showed a shift of cytoplasmic ROS in Nrf2^WT^ osteoclasts (Fig. 3*B*) to mitochondrial ROS in Nrf2^ΔΔ^ osteoclasts (Fig. 3, *B*–*E*). Our results showed that the deficiency of Nrf2 led to a reduction in mitochondrial membrane potential, as evidenced by mitotracker staining compared to control osteoclasts (Fig. 3, *F* and *G*). The results show that in the absence of Nrf2, osteoclasts undergo a metabolic reprogramming, marked by a loss of mitochondrial membrane potential and consequent increase in mitochondrial ROS production.

### Loss of Nrf2 leads to lipid peroxidation and lysosome disruption

**Figure 4:**
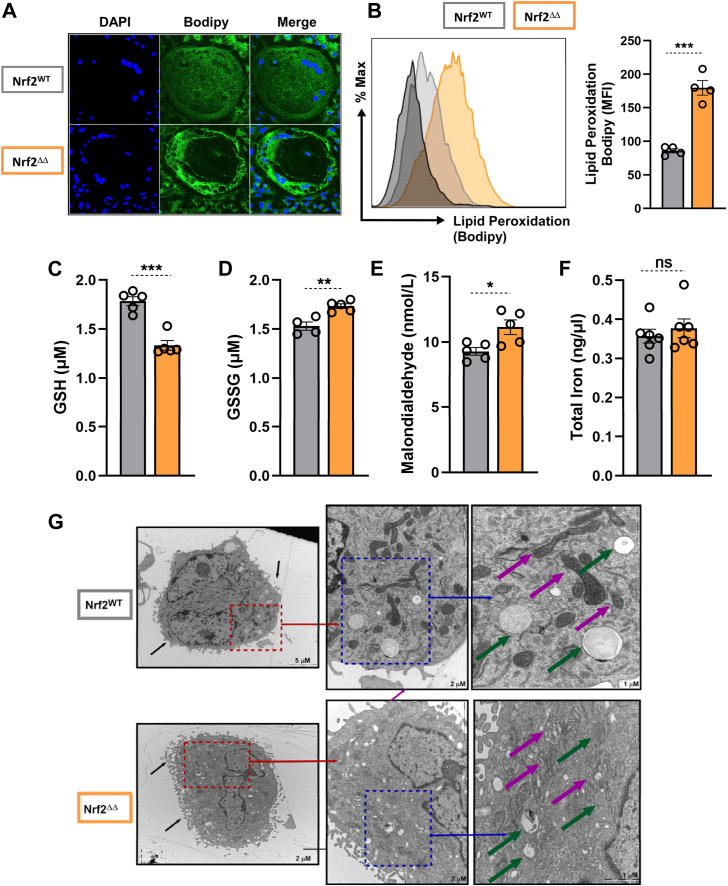
**Nrf2-deficient osteoclasts exhibit increased peroxidation of cellular, mitochondrial, and lysosomal membranes.***A*, confocal microscopy (60 × ) of mature osteoclasts from Nrf2^wt^ (*gray*) and Nrf2^ΔΔ^ (*orange*) animals treated with the Bodipy probe. Images are representative of two independent experiments. *B*, flow cytometry quantifying lipid peroxidation using the Bodipy probe in mature osteoclasts (*left*) from Nrf2^wt^ (*gray*) and Nrf2^ΔΔ^ (*orange*) animals. Histograms represent two independent experiments. Median fluorescence intensity (MFI) of Bodipy (*right*) in mature osteoclasts from Nrf2^wt^ (*gray*) and Nrf2^ΔΔ^ (*orange*) animals. Bar graph summarizes two independent experiments (n = 4). Data are presented as mean ± SEM. Unpaired two-tailed Student’s *t* test was used to calculate *p* values. ∗∗∗*p* < 0.001. *C*, fluorescence quantification of the GSH/GSSG ratio in mature osteoclasts from Nrf2^wt^ (*gray*) and Nrf2^ΔΔ^ (*orange*) animals. Bar graph summarizes two independent experiments (n = 5). Data are presented as mean ± SEM. Unpaired two-tailed Student’s *t* test was used to calculate *p* values. ∗∗∗*p* < 0.001. *D*, fluorescence quantification of reduced glutathione in mature osteoclasts from Nrf2^wt^ (*gray*) and Nrf2^ΔΔ^ (*orange*) animals. Bar graph summarizes two independent experiments (n = 5). Data are presented as mean ± SEM. Unpaired two-tailed Student’s *t* test was used to calculate *p* values. ∗∗*p* < 0.01. *E*, chemiluminescence quantification of malondialdehyde in mature osteoclasts from Nrf2^wt^ (*gray*) and Nrf2^ΔΔ^ (*orange*) animals. Bar graph summarizes two independent experiments (n = 5). Data are presented as mean ± SEM. Unpaired two-tailed Student’s *t* test was used to calculate *p* values. ∗*p* < 0.05.*F*, Fluorescence quantification of intracellular iron levels in mature osteoclasts from Nrf2^wt^ (*gray*) and Nrf2^ΔΔ^ (*orange*) animals. Bar graph summarizes two independent experiments (n = 6). Data are presented as mean ± SEM. Unpaired two-tailed Student’s *t* test was used to calculate *p* values. ns: not significant.*G*, Transmission electron microscopy analysis of mature osteoclasts from Nrf2^wt^ (*gray*) and Nrf2^ΔΔ^ (*orange*) animals. *Left*: 10 × magnification. *Right*: 30 × magnification. The *black arrow* indicates the cell membrane, the *purple arrow* indicates mitochondria, and the *green arrow* indicates lysosomes. Images are representative of 3 independent experiments.

Because mitochondrial ROS drives lipid peroxidation and membrane damage, we next investigated whether Nrf2 deficiency promotes oxidative damage to cellular lipids in osteoclasts. Our data show that the lack of Nrf2 in osteoclasts leads to the accumulation of peroxidized lipids on the cellular membrane (Fig 4, *A* and *B*), increased level of oxidized GSSG and decreased of GSH, indicating a high oxidative stress within these cells (Fig. 4, *C* and *D*). Consistently, we found higher levels of the malondialdehyde (MDA) in Nrf2^ΔΔ^ osteoclasts (Fig. 4*E*), in line with reduced GSH and increased membrane lipid peroxidation. Because lipid peroxidation can drive iron accumulation and trigger ferroptotic cell death, we next evaluated whether this phenotype was associated with changes in intracellular iron levels. However, Nrf2 deficiency did not show increased levels of intracellular iron in Bosteoclasts (Fig. 4*F*), indicating that enhanced lipid peroxidation in these cells occurs independently of iron overload.

Ultrastructural images analysis revealed alterations in membrane organization in Nrf2^ΔΔ^ osteoclasts. Compared to control, these osteoclasts exhibited prominent membrane invaginations (Fig. 4*G*, black arrow) and changes in lysosomal ultrastructure, characterized by smaller and less defined lysosomal compartments (Fig. 4*G*, green arrows). Moreover, Nrf2^ΔΔ^ osteoclasts displayed marked mitochondrial abnormalities, including reduced cristae density and disrupted cristae organization compared to controls (Fig. 4*G*, purple arrow). These results are consistent with previous results showing that Nrf2-deficient osteoclasts exhibit an impaired mitochondrial membrane potential (Fig. 3*E*). In summary, our results suggest that the deficiency of Nrf2 in osteoclasts promotes lipid peroxidation, leading to impaired lysosomal and mitochondrial membrane integrity, which in turn impacts the production and release of cathepsin K.

### The deletion of Nrf2 in mature osteoclasts leads to an increase in bone volume in adult mice

**Figure 5:**
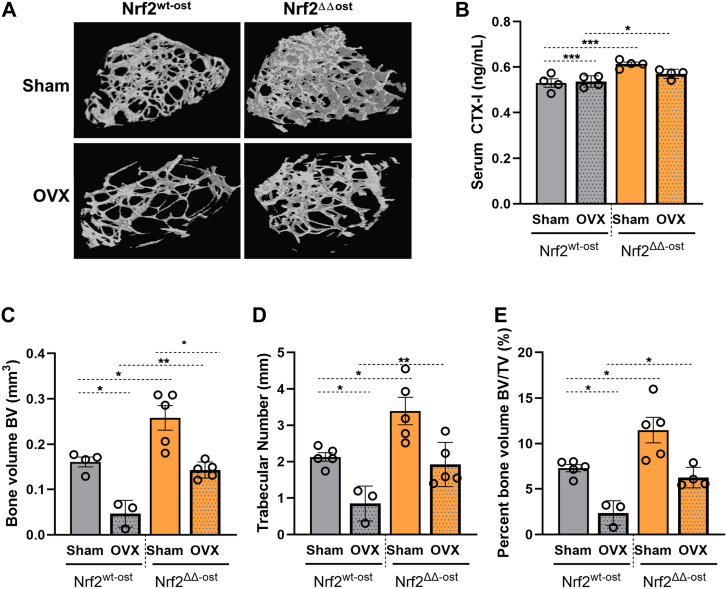
**Mice with Nrf2-deficient osteoclasts are protected against bone loss.***A*, micro-computed tomography of femurs extracted from female Nrf2^wt-ost^ and Nrf2^ΔΔ-ost^ animals that underwent ovariectomy (OVX) for 8 weeks. Animals that did not undergo OVX represent the control group (sham). Images are representative of two independent experiments (n = 3–5). *B*, ELISA quantification of serum CTX-I (ng/ml) in Nrf2^wt-ost^ and Nrf2^ΔΔ-ost^ animals. Bar graphs are representative of two independent experiments (n = 4). Two-Way Anova was used to calculate *p* values. ∗ *p* < 0.05; ∗∗ *p* < 0.01; and ∗∗∗ *p* < 0.001, ns: not significant. *C*, quantification of bone volume (BV, mm^3^) in Nrf2^wt-ost^ and Nrf2^ΔΔ-ost^ animals. Bar graphs are representative of two independent experiments (n = 5). Two-Way Anova was used to calculate *p* values. ∗ *p* < 0.05; ∗∗ *p* < 0.01. *D*, Quantification of Trabecular Number (mm^−^^1^) in Nrf2^wt-ost^ and Nrf2^ΔΔ-ost^ animals. Bar graphs are representative of two independent experiments (n = 5). Two-Way Anova was used to calculate *p* values. ∗ *p* < 0.05; ∗∗ *p* < 0.01. *E*, quantification of Percent Bone Volume (BV/TV %) in Nrf2^wt-ost^ and Nrf2^ΔΔ-ost^ animals. Bar graphs are representative of two independent experiments (n = 5). Two-Way Anova was used to calculate *p* values. ∗ *p* < 0.05.

We finally evaluated the *in vivo* relevance of our findings. We generated Nrf2-deficient mice in osteoclasts using the Ctsk^cre^ background (Nrf2^wt-ost^) to evaluate whether the deletion of Nrf2 in osteoclasts would impact bone quality in a bone loss-induced model by ovariectomy (OVX) (15). Under basal conditions, Nrf2^ΔΔost^ mice exhibited increased trabecular bone parameters, compared to controls, and were protected from OVX-induced bone loss (Fig. 5, *A*–*D*) as evidenced by a higher BV/TV, and trabecular number (Fig. 5, *A*, *C*–*E*). Consistent with these results, the level of CTX-I in the sham and OVX groups from Nrf2^ΔΔost^ mice was similar (Fig. 5*B*). Therefore, our results suggest that osteoclasts in Nrf2-deficient mice also exhibit impairments in their resorptive capacities. Overall, our results support a critical role of Nrf2 in maintaining the osteoclast function and bone resorption *in vivo*.

## Discussion

Nrf2 is a master regulator of cellular redox homeostasis, governing the transcription of numerous antioxidant response elements (AREs) that protect cells from oxidative damage (9). While its role in redox biology is well established, the role of Nrf2 in osteoclast biology remains controversial (10). Previous researchers have demonstrated that Nrf2 deficiency induces an increased osteoclast differentiation due to elevated ROS levels and intracellular accumulation of free radicals (11, 16). However, the downstream consequences of Nrf2 loss on osteoclast function and bone resorption have not been explored.

In this study, transcriptomic analyses revealed that RANKL has a significant impact on antioxidant gene expression, particularly during the early stages of osteoclast differentiation. This data suggests that ROS plays a critical role during the initial stage of osteoclastogenesis, while the subsequent activation of Nrf2 systems becomes essential to prevent oxidative stress within the cell. Despite increased differentiation, Nrf2-deficient osteoclasts exhibited a decreased level and activity of Cathepsin K, a protease required for bone resorption. This functional uncoupling between osteoclastogenesis and bone resorption highlights the crucial role of Nrf2 in regulating the specialized effector function of osteoclasts.

Evaluation of the metabolic state of osteoclasts revealed that Nrf2 deficiency profoundly disrupted redox balance, leading to mitochondrial ROS (mtROS) accumulation. The excessive production of mt-ROS promotes mitochondrial dysfunction, DNA damage, and apoptosis (17, 18, 19). Despite the increase in mt-ROS production, the viability of Nrf2-deficient cells remained unchanged, indicating resistance to death. This observation is consistent with previous reports showing that osteoclasts are resistant to the adverse effects of mt-ROS, by induction of mitochondrial antioxidant enzymes such as the enzyme superoxide dismutase 2 (SOD2) (20).

Nrf2 is a well-established regulator of lipid peroxidation and membrane redox integrity (21). In this paper, we show evidence of lipid peroxidation in osteoclasts, revealing that Nrf2 deficiency leads to pronounced oxidative damage to cellular membranes and lysosomal integrity, despite preserved osteoclast differentiation. Loss of lysosomal membrane integrity results in the leakage of lysosomal hydrolases into the cytosol, significantly affecting cell function (22). Elevated levels of malondialdehyde, a byproduct of lipid peroxidation (23), and decreased glutathione availability support the conclusion that Nrf2 is required to restrain ROS-induced lipid damage and maintain membrane homeostasis.

Lipid peroxidation has been closely linked to iron metabolism and ferroptotic cell death (24). In osteoclasts, iron overload activates Nrf2-dependent antioxidant responses, inhibiting ferroptosis and promoting osteoclastogenesis (25). NRF2 counteracts ferroptosis by activating key proteins associated with glutathione metabolism, such as MGST1 and GPX4 (26, 27). However, in our study, Nrf2-deficient cells did not express increased intracellular iron levels or activation of cell death pathways, validated by preserved ATP levels and cell viability. These findings suggest that oxidative damage driven by Nrf2 loss in osteoclasts manifests primarily as a functional impairment rather than ferroptosis or apoptosis.

Based on our *in vitro* findings, which demonstrate that NRF2 regulates redox balance, mitochondrial integrity, lipid peroxidation, and osteoclast functional maturation, we next sought to determine the *in vivo* relevance of osteoclast-intrinsic NRF2 signaling. In our vivo study, Nrf2-deficient mice, specifically in Ctsk-expressing osteoclasts, exhibited increased bone volume and trabecular number compared to WT mice, under homeostatic conditions. On the other hand, these mice are resistant to OVX-induced bone loss, indicating reduced osteoclast activity and greater protection against skeletal deterioration.

Recently, the treatment of OVX mice with different drugs, phytochemicals, or synthetic compounds with antioxidant actions has increased (28, 29, 30, 31). The results from osteoclast-deficient activity in Nrf2-deficient mice are particularly intriguing due to their novelty, as they establish the NRF2 pathway as a potential pharmacological target. Further mechanistic studies are necessary to define the specific molecular targets of NRF2 that maintain lysosomal and mitochondrial integrity and to explore the therapeutic implications of modulating NRF2activity in bone-resorptive diseases.

## Experimental procedures

### Mice

In this study, Lysm^Cre^ and Nrf2^fl/fl^Lysm^Cre^ male animals aged six to eight weeks and females Ctsk^Cre^ and Nrf2^fl/fl^Ctsk^Cre^ aged 12 to 16 weeks were used. The animals were housed with a 12-h light-dark cycle, controlled temperature, and humidity. Water and food were administered ad libitum. The Research Ethics Committee of the University of São Paulo approved the study.

### Differentiation of osteoclasts

Bone marrow cells isolated from femurs and tibias (two mice per group Nrf2^WT^ and Nrf2^ΔΔ^) were cultured in α-MEM supplemented with 1% penicillin/streptomycin and 10% fetal bovine serum (FBS) in the presence of murine M-CSF (30 ng/ml). After three days, adherent cells were seeded with M-CSF (30 ng/ml) and RANKL (10 ng/ml), and the medium was changed every three days. Cells were treated and collected according to each set of experiments. After five days, murine osteoclasts were stained, and TRAP + cells containing three or more nuclei/cell were quantified, and no distinction was made between large and small osteoclasts. Total mature osteoclasts were counted and represented as TRAP + cells/well.

### mRNA extraction and qRT-PCR

For the quantitative PCR assay, 200,000 preosteoclasts were plated in 24 wells in osteoclastogenic medium. Samples were collected at 0, 24, 48, and 72 h. Total RNA isolation was performed using the Promega SV Total Isolation System Kit (Promega) and complementary DNA was synthetized using 500 ng of mRNA and reverse transcriptase reaction kit (High-Capacity cDNA Reverse Transcription kit from Applied Biosystems; catalog no.: 4368813). The expression of genes was analyzed by TaqMan (Applied Biosystem).

### Protein extraction and western Blot

For the Western blot assay, 30 μg of protein in the RIPA buffer was quantified by BSA protocol. We used 4 to 20% Mini-PROTEAN TGX Precast Protein Gels (Biorad) and Trans-Blot Turbo Mini 0.2 μm Nitrocellulose Transfer Packs (Biorad) to transfer protein. Protein was evaluated using a nitrocellulose membrane and the primary antibodies anti-Nrf2 (Cell Signaling, 12721T), anti-Mgst1 (Abcam, ab8570), anti-Ctsk (Abcam, ab37259), and Nqo1 (Abcam, ab80588) were used. β-actin (Abcam, ab8224) was used as a housekeeping gene. The secondary antibodies used were anti-mouse (Abcam, ab6789) and anti-rabbit (Abcam, ab6728).

### scRNA-seq and bulk RNA-seq analysis

We downloaded GSE176265_non-normalized_data.txt from the GEO database (13) and used GSEA (Gene Set Enrichment Analysis) (32) software to compare the transcriptomes of osteoclasts on days 0 and 4, focusing our analysis on the Hallmark gene sets. Only FDR < 0.05 was considered enriched. We also reanalyzed single-cell RNA sequencing (scRNA-seq) of osteoclast in different days of RANKL stimulation from GSE147174 (14). The datasets were downloaded, and the RDS file was imported into the R environment (2/R Core Team, 2023) version v4.3.1 and Seurat v4.3.0. The specific criteria were applied: data pre-processing steps were filtered based on cells expressing at least 200 genes, and Feature RNA < 7000 & percent mitochondrial expression > 5 were filtered. The percentage of mitochondrial genes was regressed in the SCTransform function and Principal Component Analysis (PCA) was performed using the selected 20 major PCs and were selected for size reduction by Uniform Manifold Approximation and Projection for Dimension Reduction (UMAP). The visualization of genes illustrating expression levels was performed using R/Seurat commands (DimPlot, FeaturePlot, and DotPlot) using ggplot2 v3.4.2 and scCustomize v1.1.1 R packages. For RNA-seq analysis.

### Reabsorption assay

Murine adherent cells were seeded in 96-well hydroxyapatite-coated plates (OsteoAssay-Corning) with osteoclastogenic media. After four days, cells were removed, and the demineralized area was measured using a Stereo microscope (Leica MZ6). For resorption assay in bone slices, monocytes were seeded in culture flasks in α-MEM containing recombinant M-CSF (25 ng/ml) and RANKL (25 ng/ml). After five days, mature osteoclasts were detached and reseeded on bone disks (IDS Nordic) in the presence of M-CSF and RANKL. After three days, the discs were stained with toluidine blue. The eroded area was analyzed by light microscopy.

### CTX elisa

For the quantification of CTX-I in bone slides, supernatant and serum from ovariectomized animals, we used the IDS-iSYS CTX-I (CrossLaps) Assay Kit (IS-3000). CTX-I was analyzed using the ELISA technique following the manufacturer's recommendations.

### Flow cytometry

Osteoclasts were cultured for 72 h in a 12-well plate, followed by two washes with PBS. Subsequently, cells were incubated with accutase for 20 min at 37 °C and washed again with PBS. After centrifugation, cells were resuspended in 1 ml of PBS and stained with 5 μg/ml Hoechst 33,342, 1 μM Mitotracker Red FM (M7514 – Thermo Scientific), 1 μM Bodipy (D3861 – Invitrogen Thermo Scientific), 1 μM MitosoxRed (M36008 – Invitrogen Thermo Scientific), 1 μM CellROX (C10444 – Thermo Scientific), and 51 μM Magic Red Cathepsin K (MR-LR2 – SKU 939 Immunochemistry Technologies). The staining was performed in the dark for 30 min at 37 °C. Following incubation, cells were washed with PBS and resuspended in FACS Buffer for analysis. Cell analysis was conducted using a FACS canto two flow cytometer based on the experimental conditions.

### Confocal microscopy

BMMs were cultured in the presence of M-CSF (30 ng/ml) and RANKL (10 ng/ml) on coverslips in 24-well plates (200,000 cells per well). After three days, the cells were washed twice with PBS and fixed in 4% formaldehyde for 15 min. Cells were stained with Hoechst 33,342, Mitotracker Red CMROX (M7512 – Invitrogen Thermo Scientific), Magic Red and Bodipy for 30 min at 37 ^o^C. After staining, the cells were washed with PBS and treated with 0.05% Triton for 20 min. Following this, the cells were incubated with phalloidin Alexa 488 for 30 min. Osteoclasts were analyzed using the Leica TCS SP8 Confocal Microscope. Images were captured with 10x, 40x, and 63x objectives. Fluorescence channels A488 and A647 were used, along with the DAPI channel (450–500 nm range) for nuclear staining. The temperature was kept at 30°C throughout the time course. The images of the cells were processed using ImageJ software.

### High content microscopy

BMMs were cultured in the presence of M-CSF (30 ng/ml) and RANKL (10 ng/ml) in black-bottomed plates (10,000 cells per well). After three days, the cells were washed with PBS and incubated in the presence of MitoSOX Red and CellROX for 30 min at 37 ^o^C. The cells were then washed again and analyzed using a PerkinElmer High Content Image Screening System – Operetta.

### Transmission electronic microscopy

To analyze mitochondria, a primary culture of BM cells from Ctsk^cre/0^ and Ctsk^cre/0^Nrf2^f/f^ mice was performed, and after three days of stimulation with RANKL, the culture was fixed for two h in glutaraldehyde (2% in phosphate buffer) and included in Embed. The ultrafines were prepared and viewed using a transmission electron microscope (Jeol JEM-100 CXII equipped with a Hamamatsu ORCA-HR digital camera). Photos of 10 osteoclasts were randomly taken from the same slice.

### Viability quantification

Osteoclasts were cultured in osteoclastogenic medium, followed by treatment with test compounds. Viability was evaluated using CellTiter-Glo Reagent following manufacturer's recommendations.

### Glutathione assay quantification

The quantification of intracellular GSH and GSSG was performed using the GSH/GSSG Ratio Detection Assay Kit II (Fluorometric - Green) (ab205811). For this experiment, BMMs were cultured in the presence of M-CSF (30 ng/ml) and RANKL (10 ng/ml) for 72 h, followed by cell lysis according to the manufacturer's recommendations.

### Malondialdehyde quantification

Intracellular lipid peroxidation was quantified using the Lipid Peroxidation (MDA) Assay Kit (Sigma MAK085). BMMs were cultured in the presence of M-CSF (30 ng/ml) and RANKL (10 ng/ml) for 72 h, and the analysis was performed according to the manufacturer's recommendations.

### Iron assay quantification

Intracellular iron levels were quantified using the Iron Assay Kit (Sigma-Aldrich, MAK025). For this experiment, BMMs were cultured in the presence of M-CSF (30 ng/ml) and RANKL (10 ng/ml) for 72 h, followed by cell lysis according to the manufacturer's instructions.

### Ovariectomy model

The Ctsk^cre/0^ and Ctsk^cre/^ Nrf2^f/f^ (four females per group) mice were anesthetized, and an incision was made on each flank (right and left) to remove both side e ovarian tissues. As a control, mice were similarly manipulated, without resection of the ovarian tissue (Sham/OVX group). After a period of 30 days, the animals were euthanized, and the femurs were collected.

### Micro-CT analysis

For the three-dimensional quantitative evaluation of bone parameters in the femurs (cortical and trabecular), the femurs were dissected and fixed in 10% buffered formalin for 24 h. They were then scanned using the Micro-CT SkyScan 1172 micro-computed tomography system (Bruker Corporation, Billerica, USA), with a voxel size of 8.7 μm, 49 kV, 0.5 mm aluminum filter, and rotation angle of 0.18°. For visualization, the three-dimensional projection images (3D) were reconstructed using NRecon software (version 1.7.4.2; Skyscan; Bruker Corporation). Cross-sectional cuts of a 1 mm segment (209 sections) below the mid-diaphysis were selected for trabecular bone parameter analysis, such as trabecular bone volume fraction (BV/TV, %), trabecular thickness (Tb.Th.), and trabecular bone mineral density (g/cm^3^). Additionally, cortical region analyses were conducted, including cortical bone volume fraction (BV/TV, %), cortical thickness (Co.Th.), and cortical porosity (Ct.Po), using Data Viewer and CTAn software (Bruker micro-CT). Calibration was performed using a phantom with known hydroxyapatite density (Skyscan, Aartselaar, Belgium).

### Statistical analysis

Data are presented as mean ± SEM from at least two independent experiments. Statistical analyses were performed using GraphPad Prism 8.0.1 (GraphPad Software, Inc). Comparisons between two independent groups were conducted using an unpaired *t* test. For analysis of more than two groups on a single variable, one-way ANOVA was used. When more than two variables across two groups were analyzed, as in Figure 5, two-way ANOVA was used. A *p*-value < 0.05 was considered statistically significant. ∗*p* < 0.05, ∗∗*p* < 0.01, ∗∗∗*p* < 0.001, ∗∗∗∗*p* < 0.0001.

Grammatical and language corrections were performed using ChatGPT (OpenAI) and Grammarly, without altering the scientific content of the manuscript.

## Data availability

All data associated with this study are present in the paper. Any information related to this study is available upon reasonable request by contacting the corresponding authors.

## Supporting information

This article contains supporting information (14).

## Conflict of interest

The authors declare that they have no conflicts of interest with the contents of this article.

## Figures and Tables

**Figure S2:**